# Transformation optics with Fabry-Pérot resonances

**DOI:** 10.1038/srep08680

**Published:** 2015-03-03

**Authors:** M. M. Sadeghi, Sucheng Li, Lin Xu, Bo Hou, Huanyang Chen

**Affiliations:** 1 College of Physics, Optoelectronics and Energy, Soochow University, Suzhou 215006, China

## Abstract

Transformation optics is a powerful tool to design various novel devices, such as invisibility cloak. Fantastic effects from this technique are usually accompanied with singular mappings, resulting in challenging implementations and narrow bands of working frequencies. Here in this article, Fabry-Pérot resonances in materials of extreme anisotropy are used to design various transformation optical devices that are not only easy to realize but also work well for a set of resonant frequencies (multiple frequencies). As an example, a prototype of a cylindrical concentrator is fabricated for microwaves.

Transformation optics^1^,^2^ has become a juicy topic since it emerged in 2006, as a fantastic tool to design various devices with novel functionalities. Readers can find the progress in a recent review^3^. One of the key concerns however in this field is that, the devices designed for the intriguing effect usually come from some singular mappings. The singular material parameters are thereby required, regardless the difficulty in fabrication. Even if we can acquire some of them, the work spectra are mostly in a very narrow band^4^. By sacrificing the material parameters (therefore some of the functionalities) for non-singular cases, broadband devices are proposed and realized (e.g., the carpet cloak^5^,^6^,^7^,^8^,^9^,^10^). Therefore, we see that these two factors, fancy effect and broadband functionality, are competing against each other, bringing about quite a dilemma for the field to advance.

In this article, we will prove that Fabry-Pérot (FP) resonances can address this difficulty to some extent. Firstly, we will propose a simple one dimensional singular mapping, which can be used to design a device called "optical void". We then set equivalency between such an optical void with the one dimensional metallic slit array. The working frequencies are right at the FP resonances. With this, we can design various devices, such as a concentrator, a shifter, a rotator, a waveguide bend, and a waveguide periscope. In particular, we fabricate a prototype of a concentrator for microwave frequencies. Our approach can be used to design novel devices for a series of frequencies. However, the functionalities will be compromised if the working frequencies deviate too much from the FP resonant frequencies.

## Methods

### Theory and simulation

All the simulated field patterns and the scattering cross sections in Figure 6 are obtained using the finite element solver COMSOL Multiphysics.

### Sample fabrication

The field concentrator is a cylindrical object comprising thin iron sheets arranged into the theoretically designed pattern. The iron sheet has the thickness *0.3 mm*, and is cut into the *32 × 500 mm^2^* rectangular piece. We fabricated the total *100* pieces and assembled them to be the concentrator. Their relative positions were fixed via inserting the iron pieces into an annular Plexiglas slice with designed radial slits. By using several Plexiglas slice fixtures, we fabricated the field concentrator which has an annular cross section of 50 mm < *r*′ < 82 mm and a height of *500 mm*. To facilitate the measurement, the second concentrator with *72* iron pieces and the size 50 mm < *r*′ < 80 mm is made to be used together with the oil cylinder.

### Experimental set-up

In the experiment, the samples are placed vertically with the cylindrical axis orientated along the *z*-direction, and a horn-shaped antenna, located *~100 cm* away from the field concentrator, transmits microwaves toward the sample with the H-field polarized along the vertical direction, as depicted in Supplementary Fig. S1. To map the spatial distribution of the *H_z_* component, we employ a split ring detecting antenna which is made of a coaxial cable and has a circular loop of diameter *4 mm* and a split *1 mm*. Its *S11* spectrum is measured and shown in Supplementary Fig. S2, displaying a magnetic radiating/receiving characteristic around *10 GHz*. To detect the magnetic field inside the oil sample, the ring plane of the detector is perpendicular to the *z*-directed coaxial cable which is inserted into the oil from the top of the sample. The split-ring antenna as well as the coaxial cable is mounted on a two-dimensional translation stage and is controlled to move in the horizontal *x*-*y* plane. The scanning range covers a square of *300 × 300 mm^2^* with a spatial resolution of *2 × 2 mm^2^*, but the central area is not accessible due to the sample occupation, as marked by the dashed lines in Supplementary Fig. S1. The horn antenna and the detector are connected to an S-parameter network analyzer to obtain the magnitude and phase of the *H_z_* field.

### Measurements

Before the measuring the field patterns of the scatters or concentrators, we scan the magnitude and phase of the incident *H_z_* field in air at *9.26 GHz* in Supplementary Fig. S3(a) and (b). Supplementary Fig. S3(c) shows the real part of *H_z_* field, which has the quasi-plane wave feature with slightly curved wavefronts. The related simulated magnitude, phase, and the real part of the incident *H_z_* field are shown in Supplementary Fig. S3(d), (e) and (f). Likewise, Supplementary Fig. S4(a), (b), and (c) show the magnitude, phase, and the real part of incident *H_z_* field in air at *9.35 GHz*, while the related simulated magnitude, phase, and the real part are shown in Supplementary Fig. S4(d), (e) and (f). To get the field pattern of the bare solid Plexiglas cylinder in Fig. 5e (or Supplementary Fig. S5(c)), we scan the magnitude and phase of the *H_z_* field in Supplementary Fig. S5(a) and (b). The simulated magnitude, phase, and the real part of the *H_z_* field are shown in Supplementary Fig. S5(d), (e) and (f). Likewise, we measure the magnitude and phase of the *H_z_* field for the concentrator with the solid Plexiglas cylinder in Supplementary Fig. S6(a) and (b), which can be used to obtain the real part of the *H_z_* field in Supplementary Fig. S6(c) (or Fig. 5c). The simulated magnitude, phase, and the real part of the *H_z_* field for the concentrator are shown in Supplementary Fig. S6(d), (e) and (f). For the bare oil cylinder, the magnitude, phase, and the real part of the *H_z_* field are shown in Supplementary Fig. S7(a), (b) and (c) (or Fig. 7e), while the simulated magnitude, phase, and the real part are shown in Supplementary Fig. S7(d), (e) and (f). For the concentrator with the oil cylinder, the magnitude, phase, and the real part of the *H_z_* field are shown in Supplementary Fig. S8(a), (b) and (c) (or Fig. 7c), while the simulated magnitude, phase, and the real part are shown in Supplementary Fig. S8(d), (e) and (f).

## Results and Discussion

**Figure 1:**
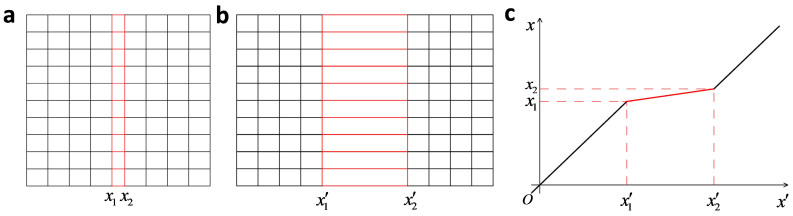
The mapping of an optical void. (a) The virtual space; (b) The physical space; (c) The detailed mathematical mapping.

Let us start with the simple mapping (see in Fig. 1c):
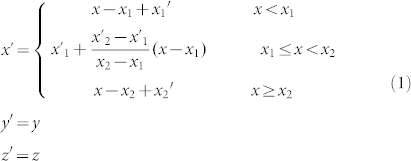
This mapping transformed a slab *x*_1_ ≤ *x* < *x*_2_ in virtual space (Fig. 1a) into a slab *x*_1_′ ≤ *x* < *x*_2_′ in physical space (Fig. 1b). Suppose the virtual space is free space, we can obtain the material tensors of the slab *x*_1_′ ≤ *x* < *x*_2_′,
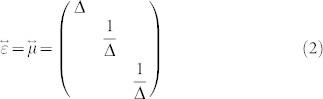
where 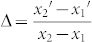. If *x*_2_ → *x*_1_, Δ → ∞, the slab *x*_1_′ ≤ *x* < *x*_2_′ functions as an optical void, which means that when wave impinges, the phase does not accumulate after passing through it.

**Figure 2:**
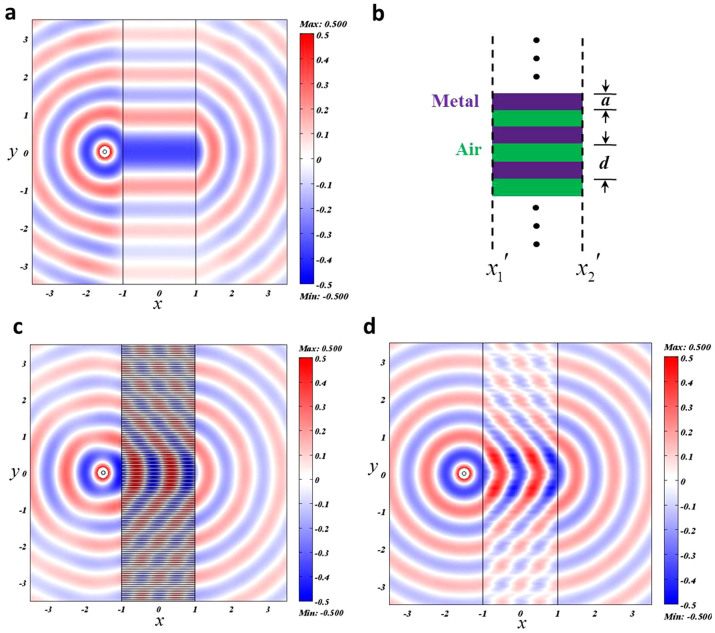
(a) The field pattern for an optical void; (b) the schematic plot of the one dimensional metallic slit array; (c) the field pattern for the one dimensional metallic slit array; (d) the field pattern for effective medium for the slit array.

As an example, we plot the field pattern for a point source setting nearby the optical void in Fig. 2a and choose the transverse magnetic (TM) polarization in this article, as we will later see that such a polarization with FP resonances has an advantage over the transverse electric (TE) polarization. Notice that all the calculations in this article are performed by the commercial FEM software COMSOL. Here only *ε_x_* = Δ and *ε_y_* = *μ_z_* = 1/Δ are required. In the simulation, we set Δ = 100000, *x*_2_′ − *x*_1_′ = 2 and the wavelength *λ* = 1. The point source is a numerical test by setting the magnetic field *H_z_* as a constant at a tiny circular boundary. From the field pattern, we find that the image of the point source is perfectly transmitted from left to right, indicating the functionality of the optical void.

Now we recall the one dimensional metallic slit array, which can support spoof surface plasmons^11^. Suppose the slit array is along *x*-direction, the width of the slits is *a*, the width of the unit cell along *y*-direction is *d*, as shown in Fig. 2b. Through the design of the perfect endoscopes (materials with extreme anisotropy)^12^,^13^,^14^,^15^,^16^,^17^,^18^,^19^,^20^,^21^,^22^,^23^, we know that when *x*_2_′ − *x*_1_′ = *mλ* (*m* = 1,2,3…), the same functionality of the optical void can be achieved. For example, as we set *d* = 2*a* = 0.1 in Fig. 2c, the slit array can perfectly transmit an image of a point source at *λ* = 1 (here *m* = 2). As *λ* ≫ *d*, the slit array can also be regarded as a slab with *ε_x_* = ∞, *ε_y_* = 2 and *μ_z_* = 0.5^11^. The same functionality of this effective medium is demonstrated in Fig. 2d (in the simulation *ε_x_* = 100000). In fact, we numerically find a general condition for such a perfect transmission from full-wave simulations, i.e.,
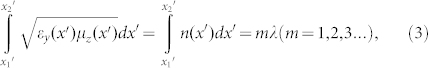
the optical path in *x*-direction is integer times of the wavelength, which is actually the FP resonance condition (for simple cases, such as a homogeneous slab, this condition can be analytically proved following Ref. 18). For TE modes, the thin slits cannot transmit waves as the working frequencies are below the cut-off frequencies.

After the above equivalency is built up between the optical void and the metallic slit array, we can use the FP resonances in transformation optics and design many devices that can be easily implemented and capable of working for a series of frequencies. To the best of our knowledge, this is the first time that FP resonances in materials of extreme anisotropy are used to design transformation media.

**Figure 3:**
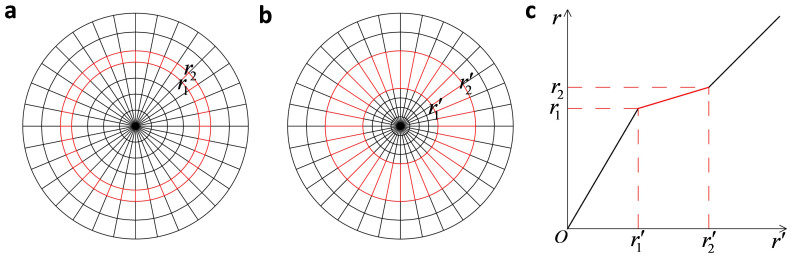
The mapping of a cylindrical concentrator. (a) The virtual space; (b) The physical space; (c) The detailed mathematical mapping.

**Figure 4:**
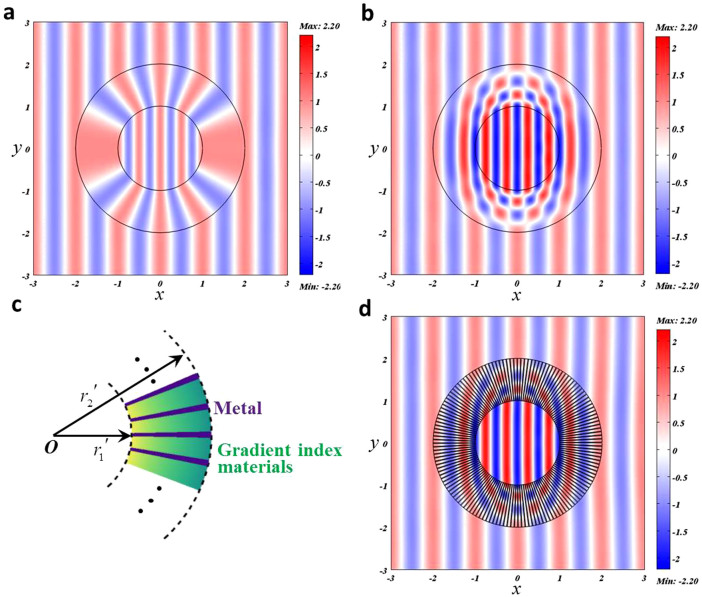
(a) The field pattern for cylindrical concentrator of the original version; (b) the field pattern for the FP version; (c) the schematic plot of the implementation by inserting thin metallic plates in a dielectric profile; (d). the field pattern for the implementation version.

For example, let us look at a similar mapping in circular cylindrical coordinate (see in Fig. 3c)^24^,
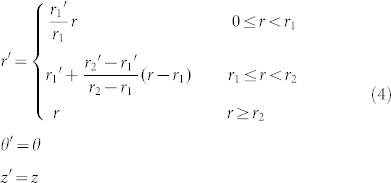
which maps a concentric cylindrical layer *r*_1_ ≤ *r* < *r*_2_ in virtual space (Fig. 3a) into another concentric cylindrical layer *r*_1_′ ≤ *r*′ < *r*_2_′ in physical space (Fig. 3b). This is a mapping to design a concentrator if we set virtual space as free space, *r*_2_′ = *r*_2_ and *r*_1_′ < *r*_1_. If *r*_1_ → *r*_2_, we shall have *ε_r_* → ∞, *ε_θ_* → 0 and *μ_z_* → 0 for *r*_1_′ ≤ *r*′ < *r*_2_′, and *ε* = 1 and 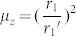 for 0 ≤ *r*′ < *r*_1_′. Figure 4a shows the perfect transparency of such a concentrator for *λ* = 1, where we set *r*_2_′ = *r*_2_ = 2, *r*_1_′ = 1 and *r*_1_ = 1.9999.

Inspired by the above FP resonance in *x*-direction, here we can also design a concentrator from FP resonance in *r*-direction. For example, we can set *ε_r_* = ∞, *ε_θ_* = *ε_θ_*(*r*′) and *μ_z_* = 1 for *r*_1_′ ≤ *r*′ < *r*_2_′, and 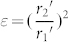 and *μ_z_* = 1 for 0 ≤ *r*′ < *r*_1_′. The FP resonance condition in *r*-direction is now numerically found as:
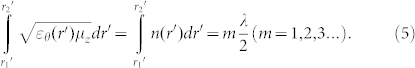
The factor 2 comes up as the waves pass the concentrator twice in *r*-direction, which is consistent with Eq. (3). Such a concentrator does not have any magnetic response and can work for a series of frequencies. To show the similar perfect transparency, we plot the field pattern in Fig. 4b for *ε_θ_*(*r*′) = (3 − *r*′)^2^ and *λ* = 1 (here *m* = 3, *r*_2_′ = 2, *r*_1_′ = 1). We note that such a version is a perfect one as the impedances match at both boundaries (*r*′ = *r*_1_′ and *r*′ = *r*_2_′). Different from the conventional concentrator (where the core medium shares the same magnetic field amplitude with the background medium), the magnetic field amplitude here is twice (in general, 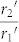) as big as that of the background medium. However, the total energy of the core medium in both cases is the same, i.e., four times (in general, 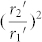) of the incident energy (a conclusion from the numerical simulations).

How can we realize such a concentrator? We can simply insert thin metallic plates along *r*-direction in a dielectric profile *ε*(*r*′) in *r*_1_′ ≤ *r*′ < *r*_2_′, see the schematic plot in Fig. 4c. For example, we insert *144* pieces of metallic plates and plot the field pattern for *λ* = 1 in Fig. 4d, where almost perfect transparency can be observed. The effect medium in *r*_1_′ ≤ *r*′ < *r*_2_′ should be modified to *ε_r_* = ∞, *ε_θ_* = *ε*(*r*′)/(1 − *f*) and *μ_z_* = 1 − *f* (*f* is the filling ratio of the inserted metallic plates). In this simulation, *f* = 0.2. If *f* is too large, impedances will mismatch at both boundaries (*r*′ = *r*_1_′ and *r*′ = *r*_2_′), causing some scattering and imperfection. We note that a similar configuration of radially arranged plates (superconducting materials plus soft ferromagnetic materials) has been proposed as a concentrator for static magnetic fields^25^. However, our work here is the first experimental attempt to realize a concentrator at finite frequencies, hence it is a breakthrough when comparing to the theoretical explorations^24^,^25^.

Now we come to implement a reduced version of the above Fig. 4c. As it is still quite tedious to realize the part between *r*_1_′ ≤ *r*′ < *r*_2_′. We make further reduction, that is, to just insert the metallic plates in air. We find from simulation that, apart from a little scattering induced by the impedance mismatching at the inner boundary *r*′ = *r*_1_′, the concentrating effect is still there. To get a perfect transparency like that in Fig. 4c, we can simply replace the dielectric core (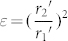 and *μ_z_* = 1) with a metamaterial core (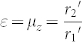). We prove such a perfect transparency analytically in Supplementary Note S1. The device can work almost perfectly for a series of FP resonant frequencies, each with a very narrow bandwidth.

**Figure 5:**
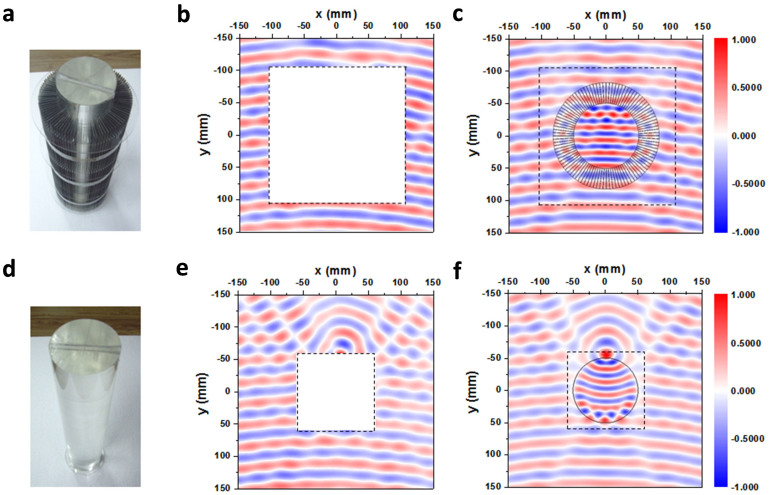
The concentrator and the effect. (a) The photo of the concentrator with the solid Plexiglas cylinder; (b) The measured H_z_ field immediately neighboring the concentrator at 9.26 GHz; (c) The simulated result; (d) The photo of the bare Plexiglas cylinder; (e) The measured H_z_ field immediately neighboring the cylinder at 9.26 GHz; (f), The simulated result.

The concentrator is assembled using the thin iron pieces, with an annular cross section of 50 mm < *r*′ < 82 mm in its final form, and is fixed surrounding a solid Plexiglas cylinder with a dielectric constant *2.7* (see in Fig. 5a). Fig. 5b is the measured *H_z_* field at *9.26 GHz*. The scanning range measures *300 × 300 mm^2^*, starting with the upper left corner, and the quasi-plane wave with slightly curved wavefronts is incident towards the -*y*-direction. The central area in the scanning region is not accessible due to the sample occupation. It is seen that the wave recovers its original wavefronts with negligible intensity decay after passing through the sample, which points out the scattering free feature of our device. To show the full picture of the *H_z_* field, we show the simulation result in Fig. 5c, where the concentrating effect is well demonstrated with a little scattering. For comparison, we also present the results for the bare Plexiglas cylinder (photo taken in Fig. 5d). The measured *H_z_* field at *9.26 GHz* is shown in Fig. 5e, and the related simulated result is shown in Fig. 5f, where we find that the scattering is enlarged. From the simulation, the dielectric core also shows a focusing effect. But with the concentrator, the wavefronts inside it become quite flat, as shown in Fig. 5c.

**Figure 6:**
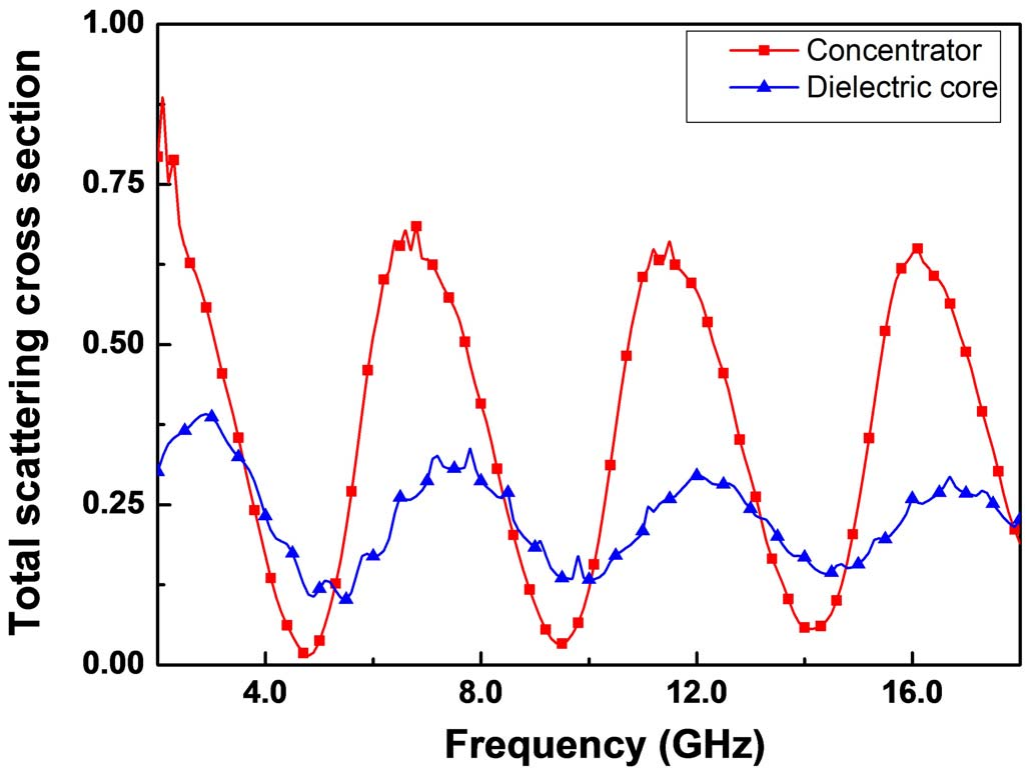
The total scattering cross sections of the bare dielectric core and the concentrator with metallic plates at different frequencies.

In fact, from the simulation, we find that the best working frequency is at *9.5 GHz*. But due to the imperfection of the sample fabrication, the working frequency is shifted to *9.26 GHz*. In Fig. 6, we plot the frequency dependences of the total scattering cross sections for the bare dielectric core and the concentrator with metallic plates. We find that the concentrator here works well for multiple frequencies. Near the resonant frequencies, the scattering cross sections are smaller than those of the bare dielectric core due to FP resonance in *r*-direction. Although the scattering at each FP resonant frequency turns bigger when compared to the perfect one in Supplementary Note S1 (due to the impedance mismatching at the inner boundary between air and dielectrics), the device here works for a broader band of frequencies.

**Figure 7:**
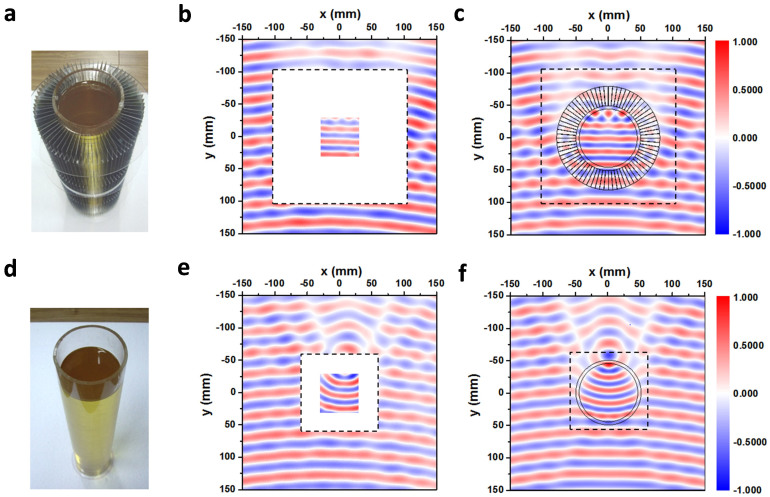
The concentrator with oil cylinder and the effect. (a) The photo of the concentrator with the oil cylinder; (b) The measured H_z_ field immediately neighboring the concentrator and inside the oil at 9.35 GHz; (c) The simulated result; (d) The photo of the bare oil cylinder; (e) The measured H_z_ field immediately neighboring the cylinder and inside the oil at 9.35 GHz; (f), The simulated result.

In order to detect the field inside the core region in the concentrator, we employed a liquid sample, as illustrated in Fig. 7a. A cylindrical Plexiglas container with the inner radius *45 mm*, the outer radius *50 mm*, and the height *53 cm* is filled with castor oil, as shown in Fig. 7d. Because the castor oil has the dielectric constant *2.6 + 0.1i*, close to the Plexiglas's value *2.7*, the oil loaded container is considered to be a liquid cylinder. The associated concentrator is fabricated using *72* iron pieces, and has an annular cross section of 50 mm < *r*′ < 80 mm in its final form. Besides scanning the exterior field, we insert the detector into the oil to sense the interior field inside the oil cylinder. A central square in the cylindrical container is now accessible to our detector. Figure 7b and 7c are the measured and simulated *H_z_* field at *9.35 GHz*, respectively. The field patterns show that the scattering is small and the wavefronts in the oil are flat, which turns out to coincide with the concentrating effect as predicted. For comparison, we also present the results for the bare oil cylinder in Fig. 7d. The measured *H_z_* field and the simulated result at *9.35 GHz* are shown in Fig. 7e and 7f, respectively. From the measured field pattern in the oil cylinder, the focusing effect is seen clearly, and is consistent with the simulated result. Due to the imperfection of the sample fabrication and the dissipation in the oil core, the working frequency is now shifted from *10 GHz* to *9.35 GHz*.

Apart from the above implementation of a reduced FP concentrator, we also design a shifter^26^, a rotator^27^, a waveguide bend^28^, and a waveguide periscope in the Supplementary Figures. In general, to apply FP resonances in transformation optics, optical voids should be designed first. Given the same principle as the above, we only numerically show the refractive index profiles and the related functionalities. Supplementary Fig. S9(a) shows the index profile of the shifter, which is composed of oblique metallic slit arrays. Supplementary Fig. S9(b) shows that the beam incident to the shifter has a displacement in *y*-direction after passing through it. By inserting specially designed curved metallic structures into an index profile in Supplementary Fig. S10(a), a perfect rotator can be implemented and the functionality is shown in Supplementary Fig. S10(b). Thirdly, we can implement devices by combining the optical conformal mapping^1^,^29^ together with the FP resonances. As the optical conformal mapping (from *z* = *x* + *iy* to *w* = *u* + *iv*) keeps the optical paths unchanged, i.e., 
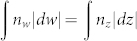
, if we insert curved metallic structures along *u*-lines (or *v*-lines), the phase changes of any waves at *v*-lines (or *u*-lines) will be unchanged, therefore all the paths have the same FP resonant frequencies. Take *w* = 2 ln *z* as an example, we can design a waveguide bend. The index profile is shown in Supplementary Fig. S11(a) (with the metallic structures along the *θ*-direction), while the related functionality is proved in Supplementary Fig. S11(b). Likewise, if we use the Zhukowski mapping, i.e., *w* = *z* + 1/*z*, a perfect one dimensional cloak can be achieved (not shown in this article). Finally, we show a perfect waveguide periscope in Supplementary Fig. S12 (a) by simply inserting a specially designed curved metallic structure in air. The functionality is demonstrated in Supplementary Fig. S12 (b). In fact, the concentrator here can also be used as a hyperlens^30^, or even find some potential application in energy harvests or wireless power transfer.

## Conclusions

In conclusion, we have shown that FP resonances can help to design various transformation optical devices, which can not only be easily implemented as they are only composed of (curved if necessary) metallic structures and dielectric profile, but can also work for a series of frequencies. As an example, we fabricate a prototype for a concentrator in microwaves and demonstrate its functionalities.

## Author Contributions

H.C. conceived the idea. H.C., M.M.S. and L.X. did the theoretical calculations and the numerical simulations. B.H., S.L. and L.X. fabricated the samples and did the experimental measurements. B.H. supervised the experimental part. H.C. supervised the whole project. H.C. and B.H. wrote the manuscript.

## Supplementary Material

Supplementary Information Supplementary Information
