# Supplementary material for: Transformation optics with Fabry-Pérot resonances
Source: Sci Rep. 2015 Mar 3;5:8680. doi: 10.1038/srep08680 (PMC4345524; doi:10.1038/srep08680)
Supplement: Supplementary Information [file srep08680-s1.pdf]

# Transformation optics with Fabry-Pérot resonances

**M. M. Sadeghi*, Sucheng Li*, Lin Xu*, Bo Hou [1], and Huanyang Chen [1]**

College of Physics, Optoelectronics and Energy, Soochow University, Suzhou 215006, China

* These authors contributed equally to this work.

[1] Correspondence and requests for materials should be addressed to H.C. (chy@suda.edu.cn) or

B.H. (houbo@suda.edu.cn)

**Supplementary Notes**

**Supplementary Note S1**

In this supplementary note, we will analytically prove that for the following configuration, perfect transparency or invisibility could be achieved at the FP resonant conditions:

$\varepsilon = \mu = 1$, for $r \ge r_2$ (Region 1);

$\varepsilon_r = \infty$ and $\varepsilon_\theta = \mu_z = 1$, $r_1 \le r < r_2$ (Region 2); (S1)

$\varepsilon = \mu_z = \frac{r_2}{r_1}$, for $0 \le r < r_1$ (Region 3).

We drop the primes here for aesthetic reasons. The magnetic field in Region 1 could be written as,

$$H_1^z(r,\theta) = \sum_n (a_n J_n(k_0 r) + b_n H_n^{(1)}(k_0 r)) e^{in\theta}, \quad \text{(S2)}$$

where $k_0 = \frac{2\pi}{\lambda}$ is the wave-vector in vacuum ($\lambda$ is the wavelength in vacuum), $b_n$ are coefficients of the scattered field to be determined, $a_n$ are coefficients of the incident field (for an incident plane wave in $x$-direction, $a_n = i^n$). $J_n$ and $H_n^{(1)}$ are the $n$-th order Bessel Functions and Hankel Functions of the first kind. As $\varepsilon_r = \infty$, the magnetic field in Region 2 shall be written as,

$$H_2^z(r,\theta) = gJ_0(k_0 r) + hY_0(k_0 r), \quad \text{(S3)}$$

with only the monopole terms ($J_0$ and $Y_0$ are the zero-th order Bessel Function and Neumann Function). $g$ and $h$ are coefficients to be determined. For Region3, the magnetic field shall be written as,

$$H_3^z(r,\theta) = \sum_n (f_n J_n(\frac{r_2}{r_1} k_0 r)) e^{in\theta}, \quad \text{(S4)}$$

where $f_n$ are coefficients of the inner field to be determined. After matching the continual boundary conditions for $H^z$ and $E^\theta$ at $r = r_1$ and $r = r_2$, we can eventually obtain,

$$b_n = a_n \frac{C_n J_n'(k_0 r_2) - D_n J_n(k_0 r_2)}{D_n H_n^{(1)}(k_0 r_2) - C_n H_n^{(1)}{}'(k_0 r_2)}, \quad \text{(S5)}$$

where

$$C_n = \frac{J_1(k_0r_1)Y_0(k_0r_2)J_n(k_0r_2) + J_0(k_0r_1)Y_0(k_0r_2)J_n'(k_0r_2) - Y_1(k_0r_1)J_0(k_0r_2)J_n(k_0r_2) - Y_0(k_0r_1)J_0(k_0r_2)J_n'(k_0r_2)}{Y_0(k_0r_1)J_1(k_0r_1) - Y_1(k_0r_1)J_0(k_0r_1)} \quad \text{(S6)}$$

$$D_n = \frac{Y_1(k_0r_1)J_1(k_0r_2)J_n(k_0r_2) + Y_0(k_0r_1)J_1(k_0r_2)J_n'(k_0r_2) - J_1(k_0r_1)Y_1(k_0r_2)J_n(k_0r_2) - J_0(k_0r_1)Y_1(k_0r_2)J_n'(k_0r_2)}{Y_0(k_0r_1)J_1(k_0r_1) - Y_1(k_0r_1)J_0(k_0r_1)} \quad \text{(S7)}$$

We thereby plot the total scattering cross section $\sigma = \sum_n |b_n|^2$ as the function of wavelength for

an incident plane wave in $x$-direction and for the case of $r_1 = 1$ and $r_2 = 2$ in Supplementary Figure S13, where we find that almost zero scattering cross section could be achieved at around $\lambda = \frac{2}{m}$ ($m = 1, 2, 3...$). If we substitute the asymptotic forms of Bessel Functions, Neumann functions, and Hankel Functions of the first kind into Equation (S5), we will get exact zero value of $b_n$ at $k_0(r_2 - r_1) = m\pi$, which is actually consistent with Equation (5) in the main text.

**Supplementary Figures**

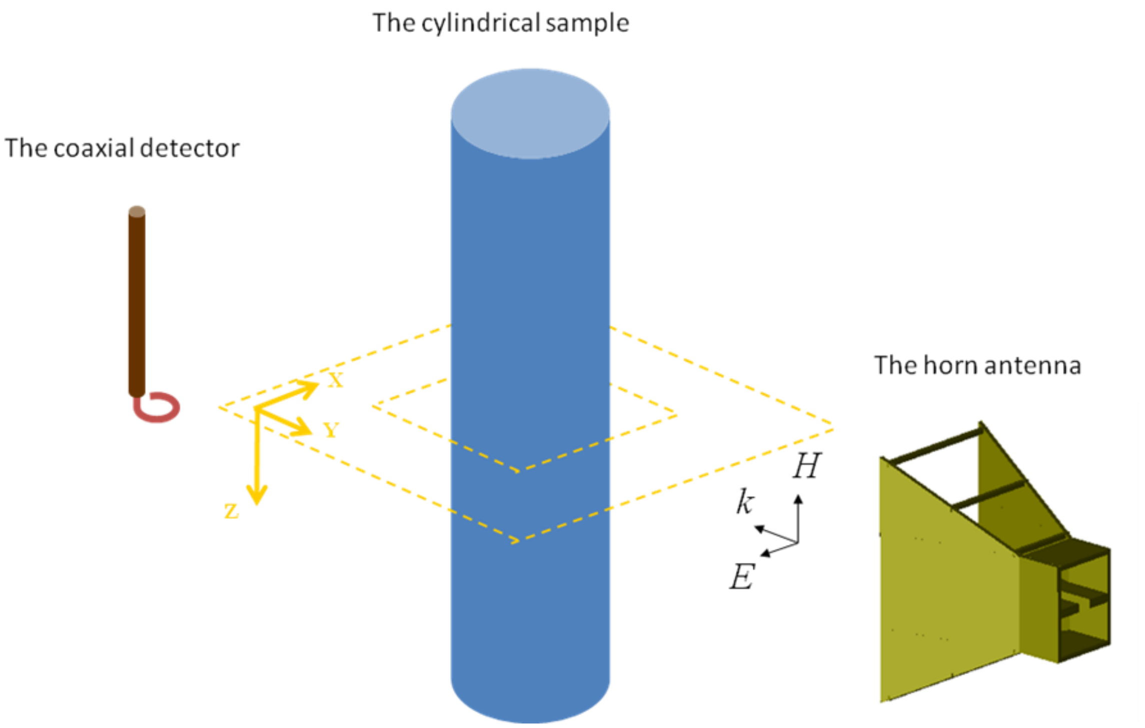


**Supplementary Figure S1. The measurement setup.** In the experiment, the samples are placed vertically with the cylindrical axis orientated along the *z*-direction, and a horn-shaped antenna, located *~100 cm* away from the samples, transmits microwaves toward them with H-field polarized along the vertical direction. The scanning plane, measuring $300\times300\ mm^2$, is located in the middle of the *500 mm* high samples to avoid the ending effect.

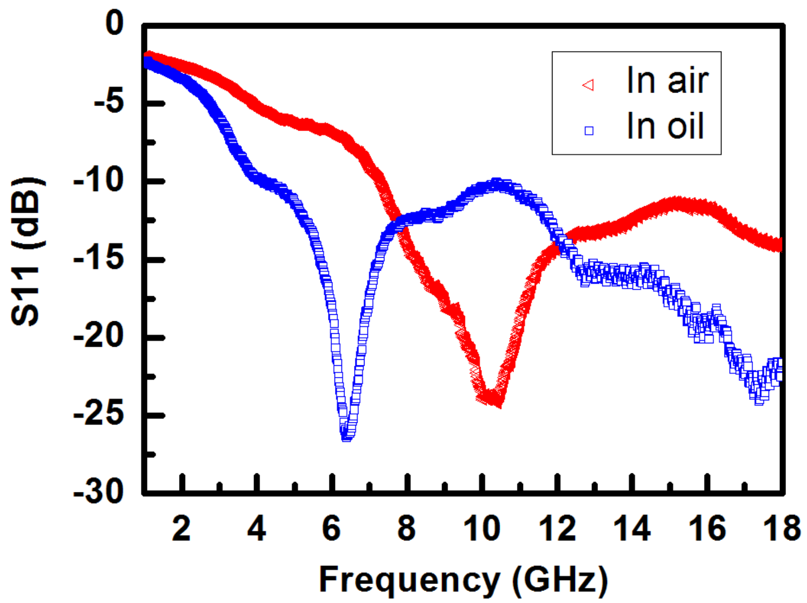


**Supplementary Figure S2. The magnetic response of the detector.** To map the spatial distribution of the $H_z$ component, we employ a split ring detecting antenna which is made of a coaxial cable and has a circular loop of diameter *4 mm* and a split *1 mm*. Its *S11* parameter is characterized first to identify the magnetic response frequency which is shown around *10GHz* in air. When the detector is inserted into the oil, the overall spectrum will downshift, bringing about a change in the radiating/receiving capability of the detector at each specific frequency, for example, one of our working frequencies (*9.35GHz*). Thus, the scanned field magnitude inside the oil cylinder needs to be corrected to make the direct comparison with the external fields in air.

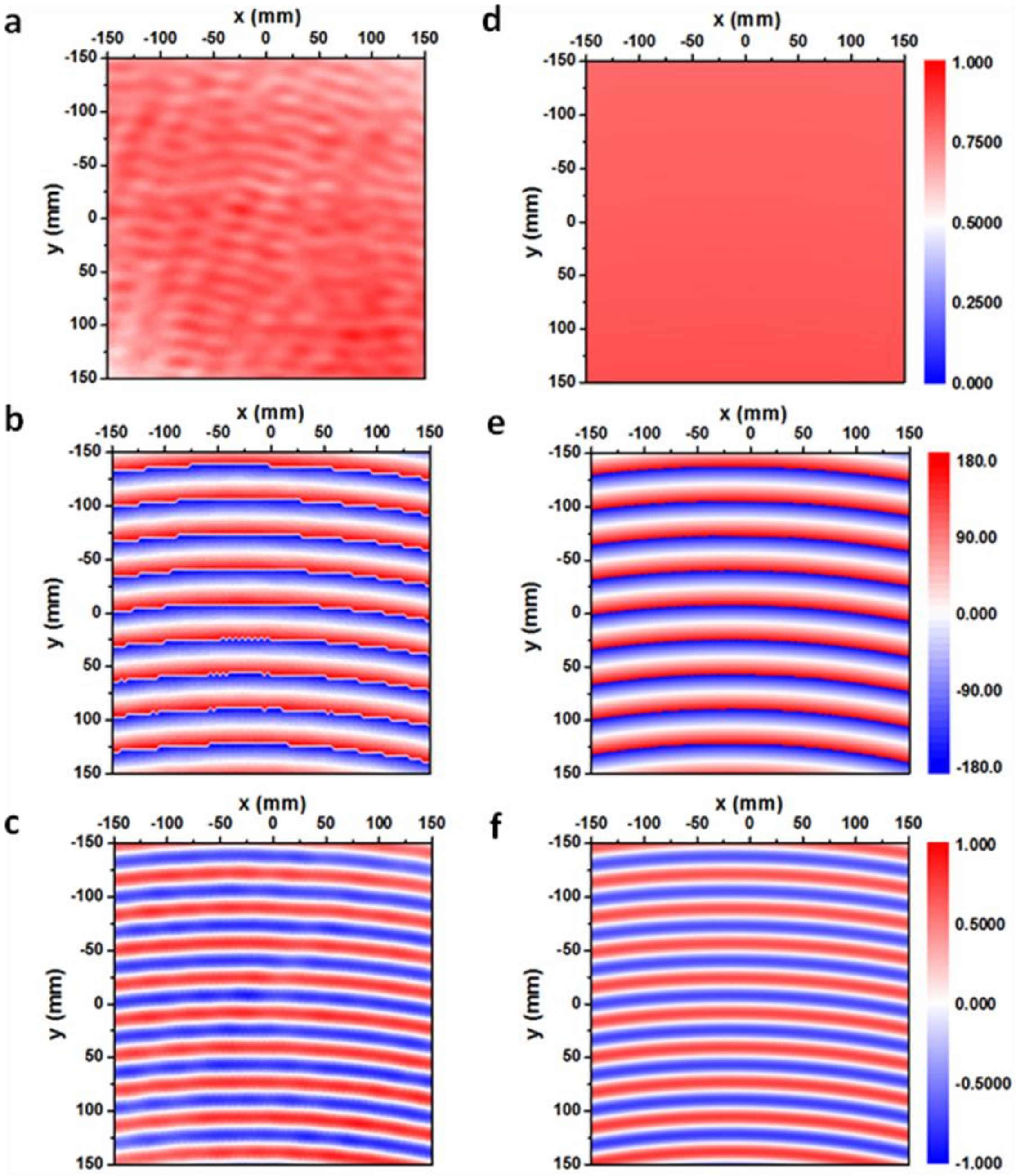


**Supplementary Figure S3. The incident H-field at *9.26GHz* in free space.** (a), The magnitude of the measured field; (b), The phase of the measured field; (c), The real part of the measured field; (d), The magnitude of the simulated field; (e), The phase of the simulated field; (f), The real part of the simulated field. It is seen that the field is incident toward the -*y* direction and has the quasi-plane wave feature with slightly curved wavefronts.

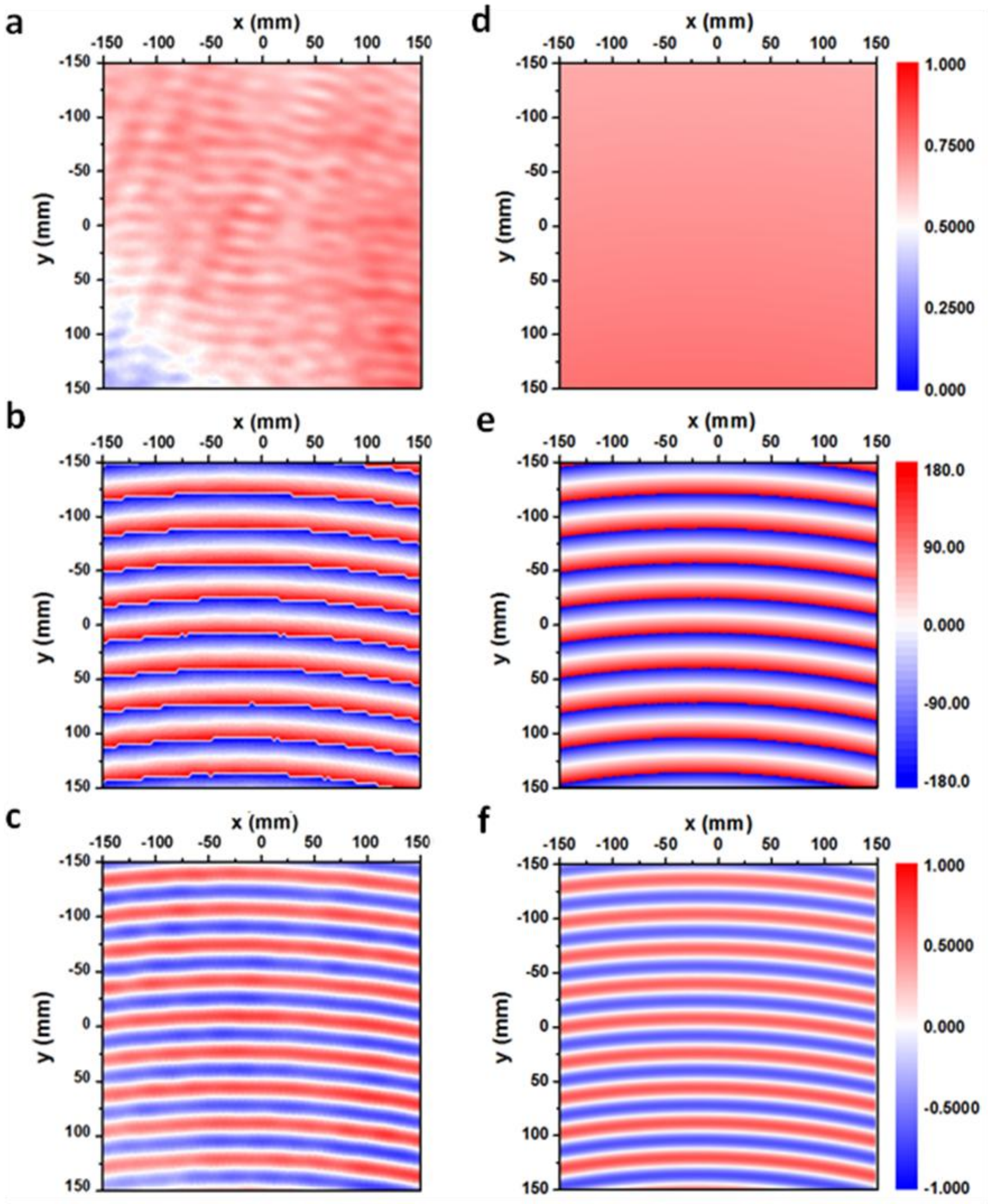


**Supplementary Figure S4. The incident H-field at *9.35GHz* in free space.** (a), The magnitude of the measured field; (b), The phase of the measured field; (c), The real part of the measured field; (d), The magnitude of the simulated field; (e), The phase of the simulated field; (f), The real part of the simulated field.

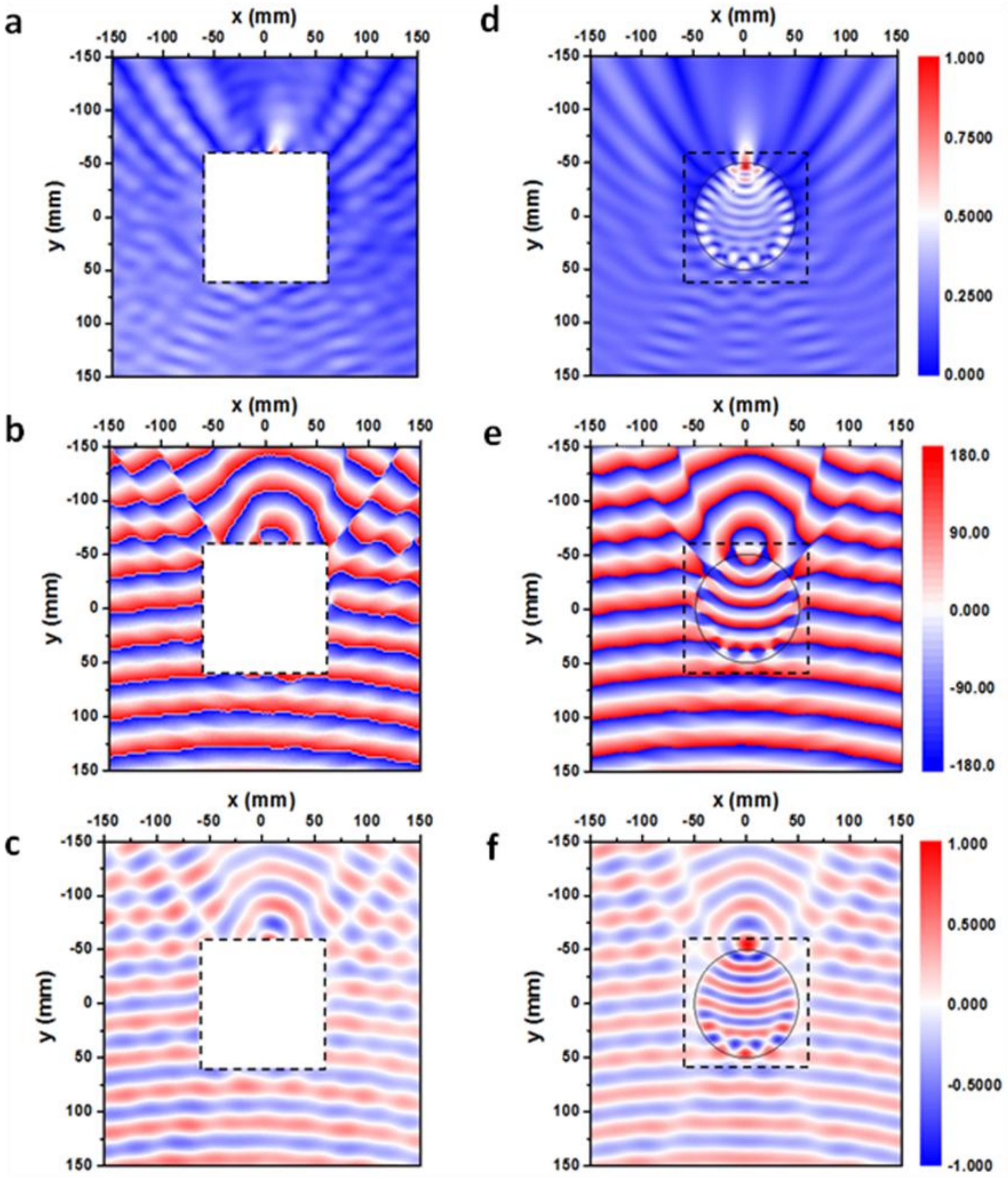


**Supplementary Figure S5. The H-field of the solid Plexiglas cylinder at *9.26GHz*.** (a), The magnitude of the measured field; (b), The phase of the measured field; (c), The real part of the measured field; (d), The magnitude of the simulated field; (e), The phase of the simulated field; (f), The real part of the simulated field.

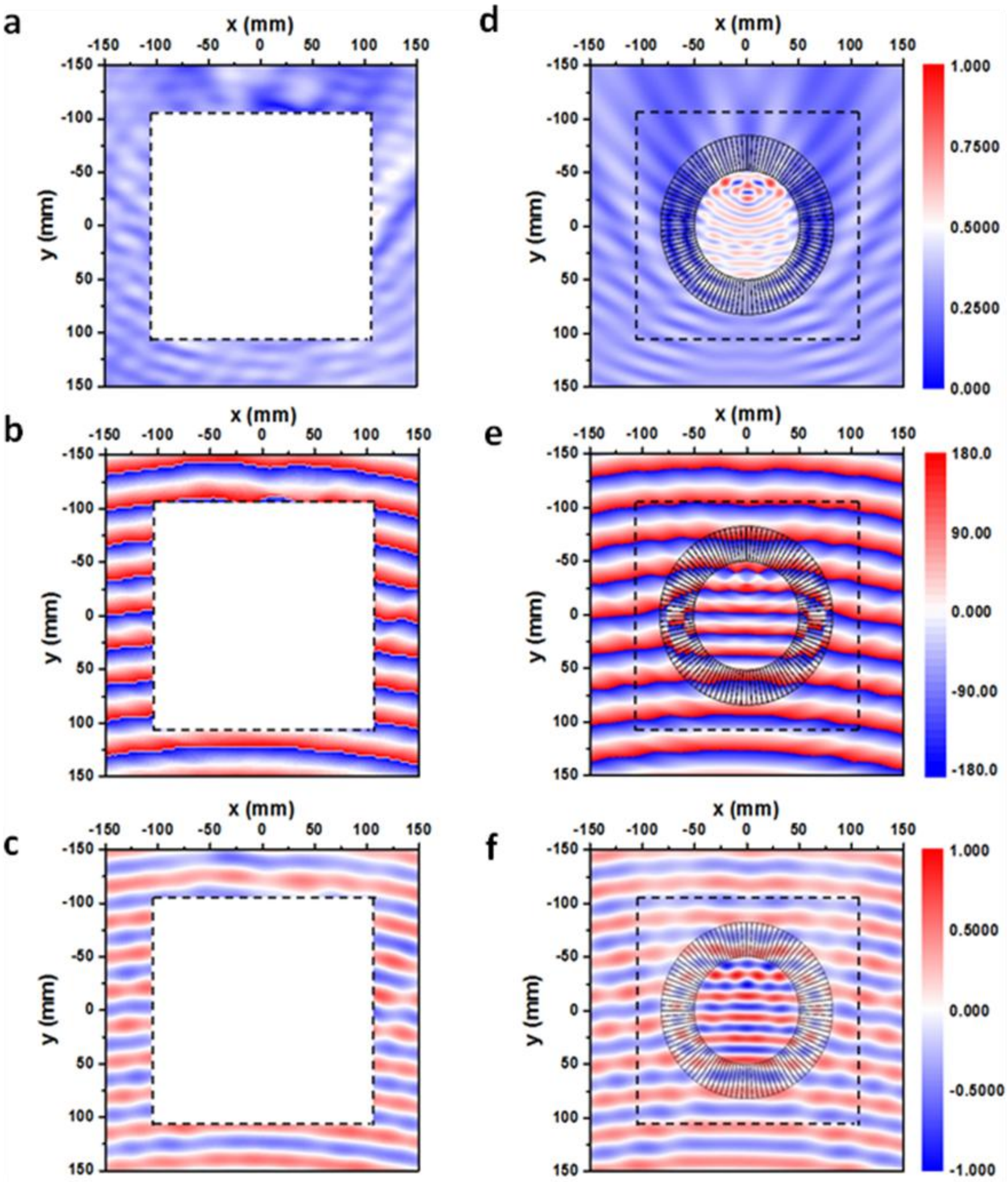


**Supplementary Figure S6. The H-field of the concentrator with the solid Plexiglas cylinder at *9.26GHz.*** (a), The magnitude of the measured field; (b), The phase of the measured field; (c), The real part of the measured field; (d), The magnitude of the simulated field; (e), The phase of the simulated field; (f), The real part of the simulated field.

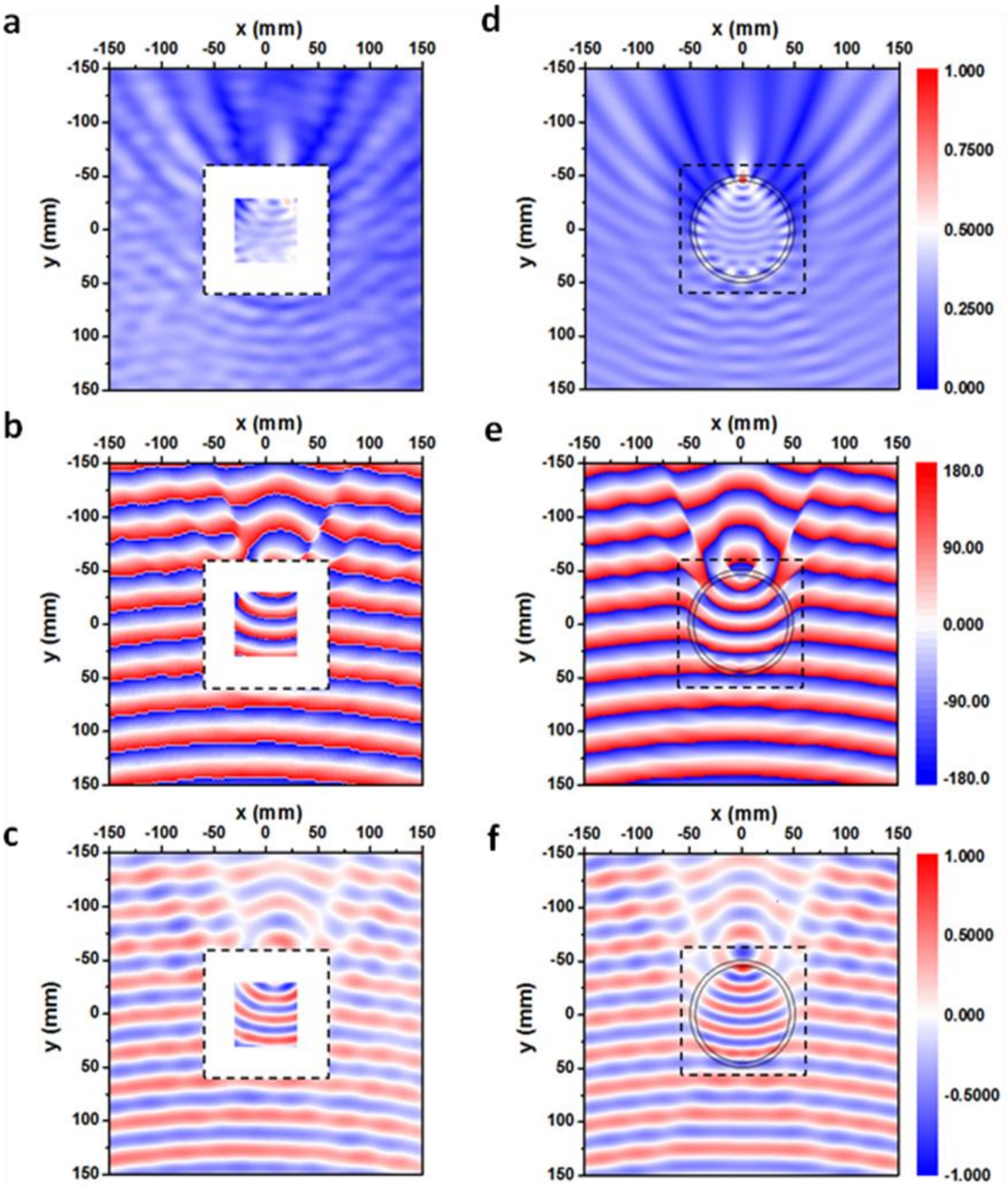


**Supplementary Figure S7. The H-field of the oil cylinder at *9.35GHz.*** (a), The magnitude of the measured field; (b), The phase of the measured field; (c), The real part of the measured field; (d), The magnitude of the simulated field; (e), The phase of the simulated field; (f), The real part of the simulated field.

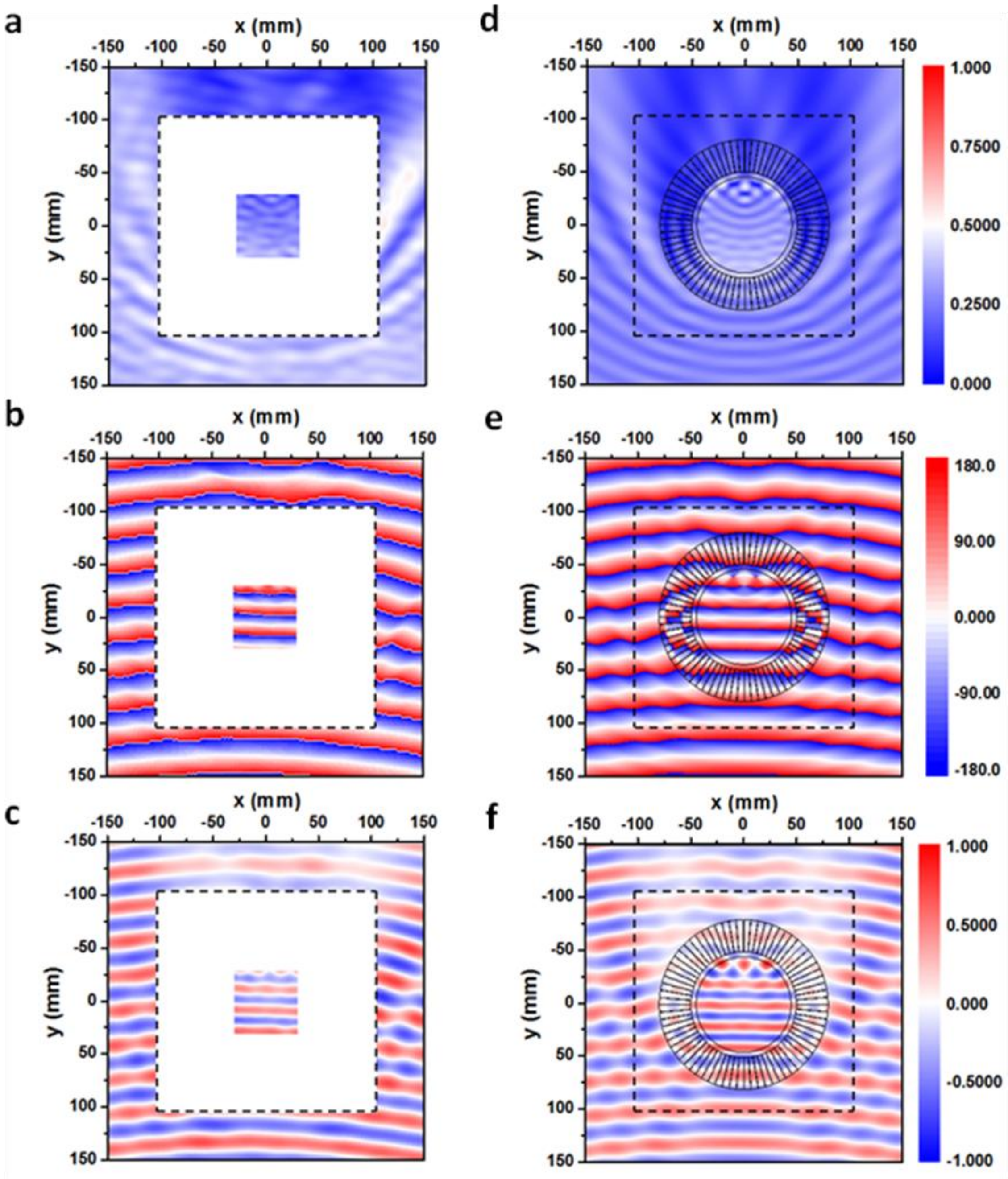


**Supplementary Figure S8. The H-field of the concentrator with the oil cylinder at *9.35GHz.*** (a), The magnitude of the measured field; (b), The phase of the measured field; (c), The real part of the measured field; (d), The magnitude of the simulated field; (e), The phase of the simulated field; (f), The real part of the simulated field.

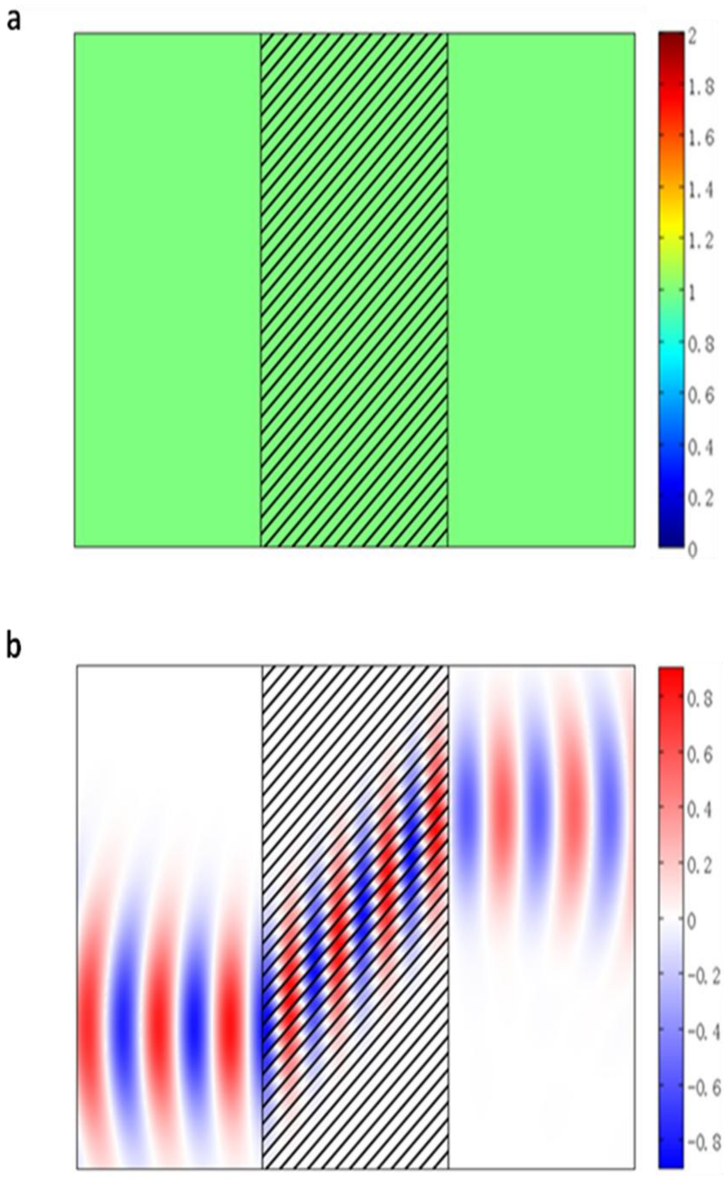


**Supplementary Figure S9. The shifter with FP resonance.** (a), The refractive index profile; (b), The shifting functionality.

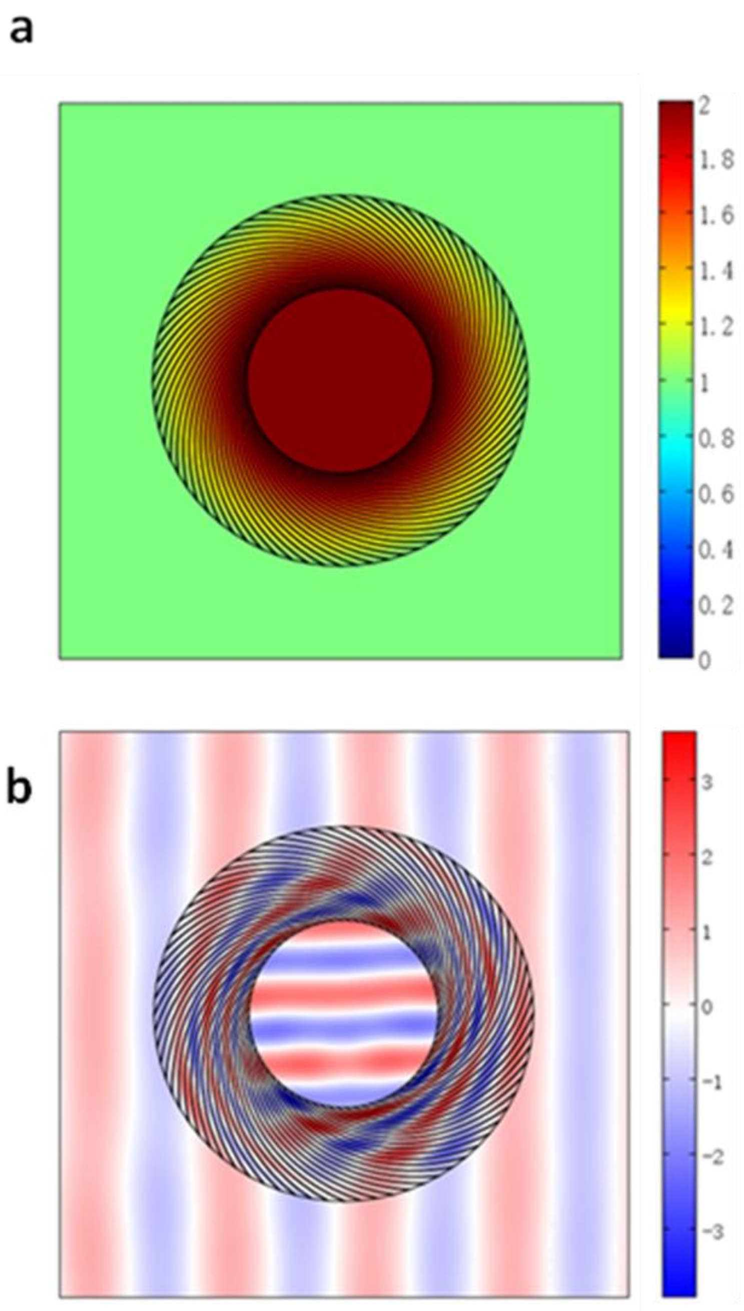


**Supplementary Figure S10. The rotator with FP resonance.** (a), The refractive index profile; (b), The wavefront rotation functionality.

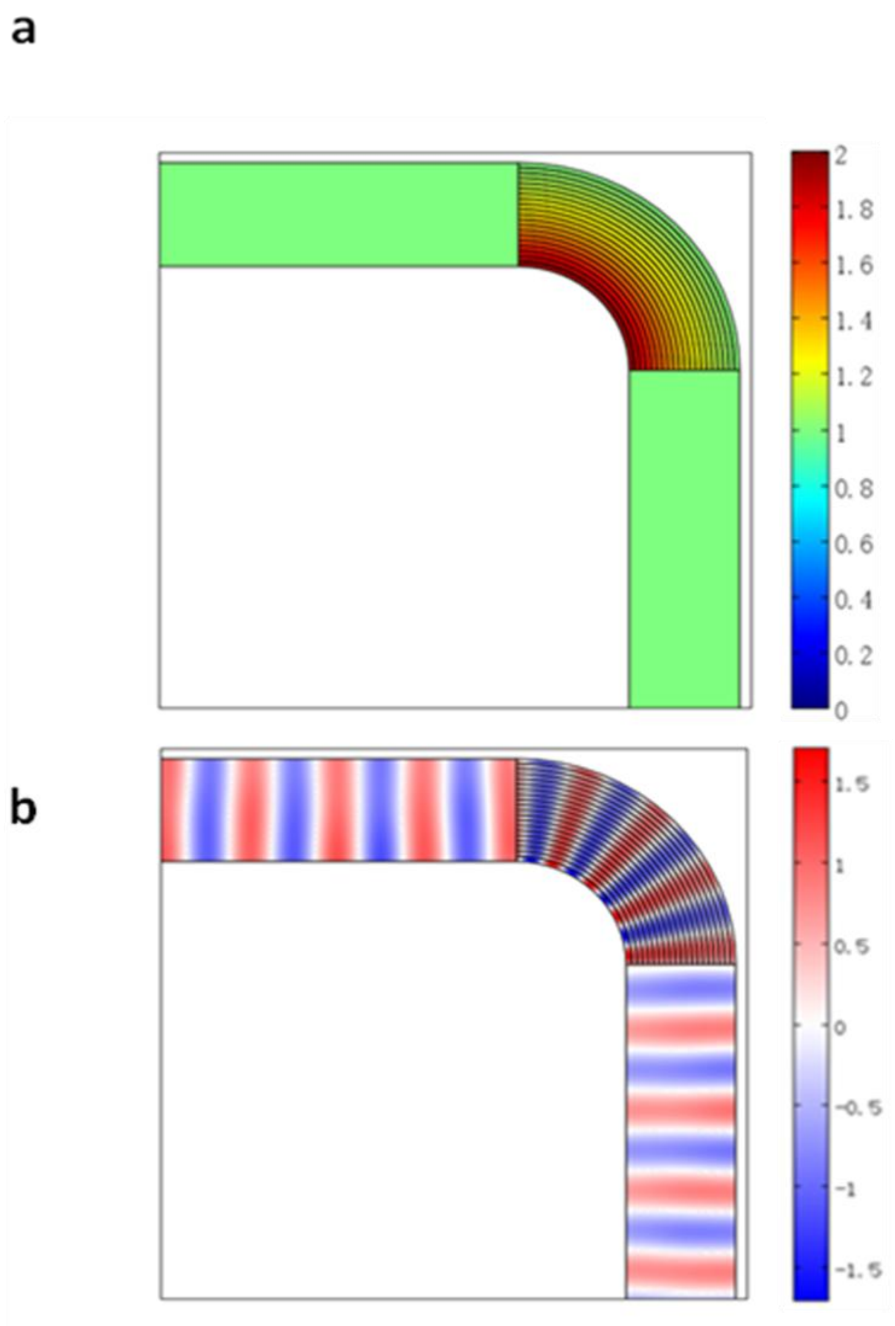


**Supplementary Figure S11. The waveguide bend with FP resonance.** (a), The refractive index profile; (b), The wave bending functionality.

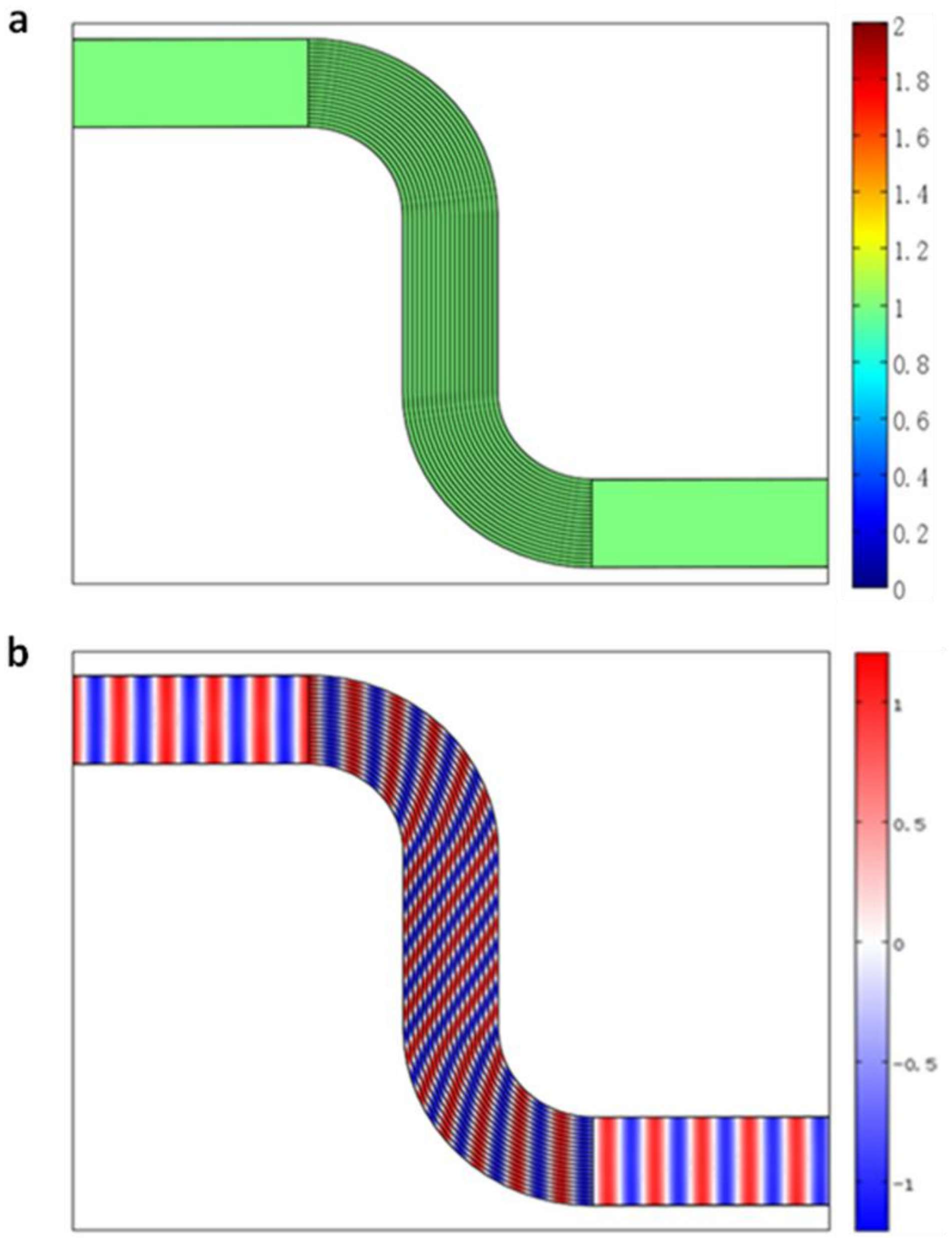


**Supplementary Figure S12. The waveguide periscope with FP resonance.** (a), The refractive index profile; (b), The periscope functionality.

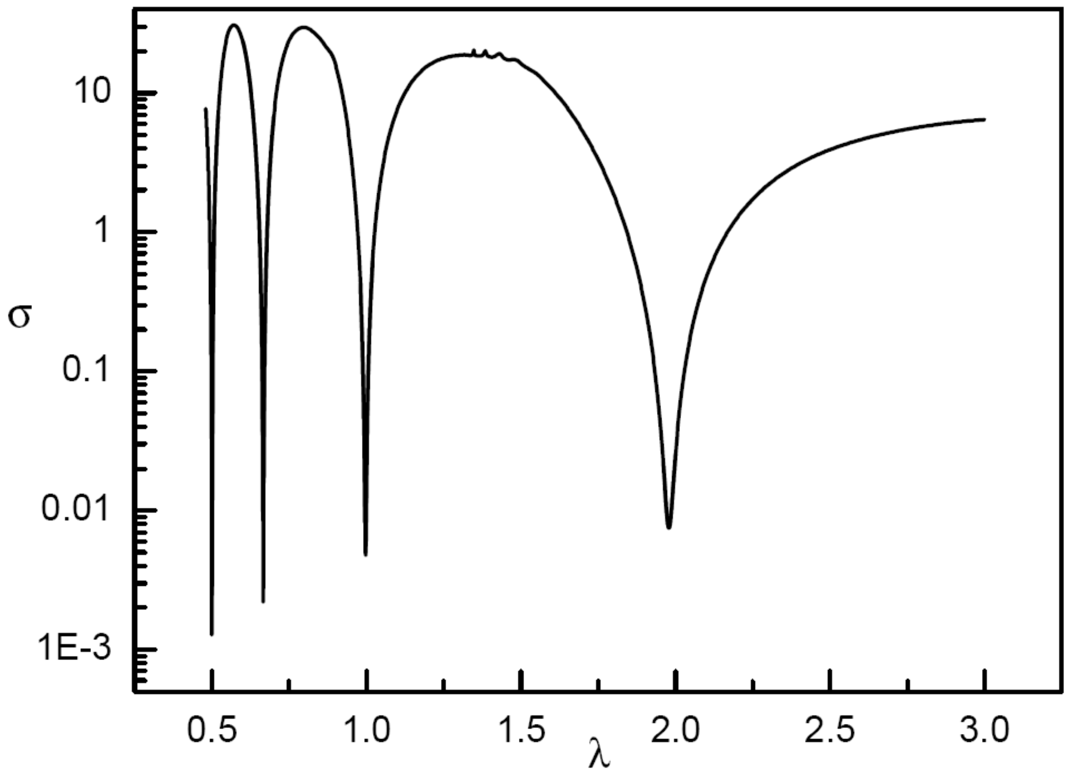


**Supplementary Figure S13. The total scattering cross section as the function of wavelength.**
